# UP-Fall Detection Dataset: A Multimodal Approach

**DOI:** 10.3390/s19091988

**Published:** 2019-04-28

**Authors:** Lourdes Martínez-Villaseñor, Hiram Ponce, Jorge Brieva, Ernesto Moya-Albor, José Núñez-Martínez, Carlos Peñafort-Asturiano

**Affiliations:** Facultad de Ingeniería, Universidad Panamericana, Augusto Rodin 498, México, Ciudad de México 03920, Mexico; jbrieva@up.edu.mx (J.B.); emoya@up.edu.mx (E.M.-A.); 0169723@up.edu.mx (J.N.-M.); 0184404@up.edu.mx (C.P.-A.)

**Keywords:** fall detection, multimodal dataset, machine learning, vision, healthcare

## Abstract

Falls, especially in elderly persons, are an important health problem worldwide. Reliable fall detection systems can mitigate negative consequences of falls. Among the important challenges and issues reported in literature is the difficulty of fair comparison between fall detection systems and machine learning techniques for detection. In this paper, we present UP-Fall Detection Dataset. The dataset comprises raw and feature sets retrieved from 17 healthy young individuals without any impairment that performed 11 activities and falls, with three attempts each. The dataset also summarizes more than 850 GB of information from wearable sensors, ambient sensors and vision devices. Two experimental use cases were shown. The aim of our dataset is to help human activity recognition and machine learning research communities to fairly compare their fall detection solutions. It also provides many experimental possibilities for the signal recognition, vision, and machine learning community.

## 1. Introduction

According to the World Health Organization (WHO), falls are, globally, the second leading cause of unintentional injury and death. Falls also frequently cause functional dependencies in elderly. “Approximately 28–35% of people aged of 65 and over fall each year increasing to 32–42% for those over 70 years of age” [1]. The incidence of falls varies in different countries and is less frequent in developed countries [2]. In Mexico, 33.5% of the elderly over 60 years of age suffered at least one fall in the year prior to the interview [3].

Fall prevalence increases with age globally and is actually considered an important health problem. Falls often require immediate medical attention since they lead to 20–30% of mild to severe injuries [1] or even death. Fall detection systems alert when a fall occurs mitigating its consequences. Negative consequences of falls can be reduced with real-time fall detection improving the time required for the patient to receive medical attention [4]. Patients sometimes remain laying in the floor causing additional medical and psychological problems if falls are not detected quickly. When monitoring falls in subjects in real conditions at less-frequent periods of time, participants tend to forget the exact data of a fall. This recall problem is more critical particularly in elder or impaired participants [5]. Fall detection systems can help to determine the real time of fall.

There are three main approaches reported in literature for fall detection systems [6] depending on whether data is acquired with wearable sensors, ambient sensors or vision devices. Igual et al. [4] categorized fall detectors into two broad approaches: context-aware systems and wearable devices. Context-aware systems consider all systems using sensors deployed in the environment, which include ambient sensors as infrared, floor, radar, microphones, and pressure sensors as well as vision-based devices. Cameras, motion capture devices, and Kinect are considered also as context-aware systems. Wearable sensors with accelerometers and gyroscopes are frequently used in fall detectors. Lately, sensors embedded in smart phones, smart watches and other portable devices have gained popularity in fall detection systems due to the great affordability and global adoption of these devices. Other authors, such as Mubashir et al. [6], divided the approaches for fall detection in three categories: wearable device-based, ambience sensor-based, and vision-based. These reviews of fall detection systems and more recent surveys like that in [7] present detailed analysis of benefits and limitations of these approaches, and additional novel multimodal systems which include different combinations of wearable, vision and ambient sensors. Among the important challenges and issues reported by most authors are privacy concerns, obtrusiveness and operative device limitations, and difficulty of comparison among techniques. This last issue is caused by the lack of public databases, especially those recording real falls of elderly.

Due to infrequency and diversity of falls in real life, it is difficult to collect datasets of actual unexpected falls. Most datasets for fall detection are simulated in laboratory settings. Khan et al. [8] described the problem of the enormous imbalance of real falls data: if all persons in a nursery where to fall 2.6 times on average in a year, the final dataset recorded in one year would have 31.55 million of normal activities per person and 2.6 falls. Therefore, although simulated fall data cannot really reproduce a fall exactly, building datasets collecting data of volunteers that simulate different falls seems still the best option for fall detection system evaluation. Many surveys [8,9] reported that there is a lack of reference framework and a few publicly available datasets for fall detection can be found. These facts, in addition to almost no access to real data, hinder systems and method validation and comparison.

We present a publicly available multimodal dataset for fall detection in order to address the aforementioned problem. The UP-Fall Detection dataset was collected using 17 healthy young subjects without any impairment using multiple modalities namely wearable sensors, ambient sensors and vision devices. The volunteers performed six daily living activities and simulated five different types of falls, with three attempts each. We use five wearable sensors to collect accelerometer, gyroscope and ambient light data. In addition, we acquired data from one electroencephalograph (EEG) headset, six infrared sensors, and two cameras. This dataset comprises raw and feature sets summarizing more than 850 GB of information from wearable sensors, ambient sensors and vision devices.

The aim of our dataset is to help human activity recognition and machine learning research communities to fairly compare their fall detection solutions. It also provides many experimental possibilities for the signal recognition, vision, and machine learning community. We are aware that as the falls were simulated by young healthy adults without impairment for safety reasons, some differences can be found with real falls in elderly. Nevertheless, this dataset can be used to transfer learning experiments for prediction in elderly people or adults with impairments.

Two experimental use cases were presented in this paper: Modalities configuration and benchmark of machine learning models.

The rest of the paper is organized as follows: firstly, an overview of fall detection datasets is presented in Section 2. Secondly, our UP-Fall Detection Dataset is described in Section 3. We explain two experimental use cases in Section 4. Experiments and results are presented in Section 5. We discuss our results and present conclusions in Section 6 and Section 7, respectively.

## 2. Databases for Fall Detection

There are many fall detection systems reported in literature, hence very few datasets are publicly available. In this section, we present an overview of fall detection datasets. We considered sensor-based databases those including wearable or ambient sensors; vision-based databases those including regular or depth cameras or motion capture data; multimodal databases those containing a combination of sensors and/or cameras. There are some important publicly available datasets for human activity recognition like SCUT-NAA [10] that are excluded from this overview since they do not include falls.

### 2.1. Wearable-Based Databases

The most cited datasets for fall detection-based sensors are reported in [11,12,13,14,15], and they are summarized in Table 1.

DLR (German Aerospace Center) dataset [11] is the collection of data from one Inertial Measurement Unit (IMU) worn in the belt of 16 people (6 female and 5 male) whose ages ranged from 23 to 50 years old. They consider seven activities (walking, running, standing, sitting, laying, falling and jumping). The types of fall were not distinguished.

MobiFall fall detection dataset [12], was developed by the Biomedical Informatics and eHealth Laboratory of Technological Educational Institute of Crete. They captured data generated from inertial-sensors of a Smartphone (3D accelerometer and gyroscope) positioned in trousers pocket. The 24 subjects, seventeen male and seven female with an age range 22–47 years performed between 3 to 6 trials for each activity. The authors considered four types of falls and nine different activities of daily living (ADL).

The tFall dataset developed by EduQTech (Education, Quality and Technology) in Universidad de Zaragoza [13] collected data from ten participant, three female and seven male, with age ranged from 20 to 42 years old. They obtained data from two smartphones carried by the subjects in everyday life for ADL. The subjects simulated eight types of common falls among elderly coded as FALL and daily living activities coded as ADL.

Vilarinho et al. [14] gathered information from a smartphone carried in the thigh pocket and a smartwatch worn on the wrist to create Project gravity dataset. Three young participants, ranged age 22 to 32, performed seven ADL activities and twelve types of fall done simulating natural ADL and a sudden fall. They combine threshold and machine learning techniques for fall detection.

UMAFall [16] is a dataset including three types of falls and eight ADL obtained from a smartphone worn in right thigh pocket and four wearable sensors worn in ankle, waist, right wrist and chest. Subjects executed at least three trials of each activity in a domestic environmental. They used a threshold-based approach for fall detection.

SisFall is a dataset [15] of falls and ADL obtained with self-developed embedded device with two accelerometers and one gyroscope. The device was positioned the waist. The dataset was generated with the collaboration of 38 participants with 15 elderly people and 23 young adults from ranged age 19 to 75 years old. They selected 19 ADL activities and 15 interesting types of fall simulated when doing another ADL activity. It is important to notice that this dataset is the only including elderly in their trials.

These datasets only include wearable sensors, commercial, self-developed or embedded in smart devices, especially in smart phones. Only a few authors use only near field image sensor [17], Pressure and infrared sensors [18] or only infrared sensors [19]. To our knowledge, no dataset is publicly available with ambient sensors or other type of sensors for fall detection.

### 2.2. Vision-Based Databases

The vision-based approaches can be based in normal RGB camera or web camera, and depth camera such as Kinect. Motion capture cameras are also used for fall detection. RGB cameras major issues are privacy, occlusion, and illumination. The use of Kinect for fall detection has been increased given that it can obtain 3D information by tracing a human [7]. Hence, the Kinect cannot cover an entire room because the resolution decreases in the depth image hindering fall detection.

SDUFall [20] built a public dataset with one Kinect camera including five daily living activities and falls performed by ten young women and men. Actions are simulated and they include some changes such as carrying/not carrying an object, light on/off, changes of position and direction relative to the camera. Although it was publicly available sometime, it cannot be found anymore.

Zhang et al. [21] presented two datasets recorded with two Kinect cameras simultaneously from two different points of view. The first dataset (EDF) ten subjects performed two falls for each of eight directions in each point of view. They also recorded five more different actions that could be similar to falling: picking up something, sitting in the floor, laying, tying shoe laces, do plank exercise. The second dataset (OCCU) focused on collecting occluded falls also with two Kinect cameras. Five subjects performed 60 occluded falls and similar different actions as in the first dataset.

Charfi et al. [22] presented video sequences using a single RGB camera in four different locations containing falls, normal activities. This dataset present sequences in four different locations and falls in different directions. It also includes variances to provide examples of main issues: illumination variances, occlusions, cluttered and textured background.

Mastorakis et al. [19] dataset was collected with a Microsoft Kinect placed at a height of 2014 cm inclined to the floor plane. They captured information of eight subjects which performed 48 simulated falls (backward, forward and sideways), 32 sitting, 48 laying, 32 picking up an item and other activities. Two subjects performed the activities in slow motion imitating an elderly person.

Other interesting vision-based datasets have been reported in literature, but they are not publicly available to our knowledge. Auvinet et al. [23] presented a dataset for fall detection built acquired with an eight camera system simulating falls and normal activities by one subject. Different types of falls were recorded namely: Forward fall, backwards fall, fall when sitting down, loss of balance. All of these falls were identified with one class: falling. Walking, standing up, laying, crouching, moving down, moving up, sitting, laying on a sofa, and moving horizontally are the daily living activities recollected in this dataset. An extensive dataset recollected with Microsoft Kinect was presented in [24]. They collected data of 16 residents in homes for older people; gathering 454 falls (445 simulated and 9 real falls), standing, sitting, and laying down positions. Table 2 summarizes the vision-based datasets for fall detection.

### 2.3. Multimodal Databases

The UR (University of Rzeszow) fall detection dataset [26] was generated recollecting data from an IMU inertial device connected via Bluetooth and 2 Kinects connected via USB. Five volunteers were recorded doing 70 sequences of falls and ADL. Some of these are fall-like activities in typical rooms. There were two kinds of falls: falling from standing position and falling from sitting on a chair. Each register contains sequences of depth and RGB images for two cameras and raw accelerometer data. The authors used a threshold-based fall detection method.

Multimodal Human Action Database (MHAD) [27] presented by [28] contains 11 actions performed by 12 volunteers (7 male and 5 female). Although the dataset registered very dynamic actions, falls were not considered. Nevertheless, this dataset is important given that actions were simultaneously captured with an optical motion capture system, four multi-view cameras arranged in four clusters, one Kinect system, six wireless accelerometers, and four microphones. Table 3 summarizes these databases in comparison with our proposed database.

Dovgan et al. [25] presented a prototype system that detects falls and behavior changes for elderly care. They performed three test and recollected data from normal activities, falling and imitations of several health problems. The first experiment collects data from Smart sensor system at 10 Hz with 12 tags attached to the wrists, elbows, shoulders, hips, knees and ankles. A comparison dataset was created with Ubisense sensor system and with an Xsens accelerometer. Four Ubisense tags were attached to the waist, chest ankles, and one accelerometer worn on the chest of 10 individuals. For the third test four persons used only the Ubisense system. Four types of falls, four health problems, and ADL were imitated in these experiments. We describe details of these datasets in Table 2 and Table 3.

## 3. UP-Fall Detection Dataset

This section presents the UP-Fall Detection dataset and describes the process of its acquisition, pre-processing, consolidating and storaging. In addition, one possible feature extraction process is also reported.

### 3.1. Description of the Dataset

We present a large dataset mainly for fall detection, namely UP-Fall Detection, that includes 11 activities and 3 trials per activity. Subjects performed six simple human daily activities as well as five different types of human falls. These data were collected over 17 healthy young adults without impairment using a multimodal approach, i.e., wearable sensors, ambient sensors and vision devices. The consolidated dataset (812 GB), as well as, the feature dataset (171 GB) are publicly available in http://sites.google.com/up.edu.mx/har-up/.

The data were collected over a period of four weeks, from 18 June to 13 July 2018 in the third floor of the Faculty of Engineering, Universidad Panamericana, Mexico City, Mexico. All the devices and equipment for measurements were connected locally to a set of computers. These computers centralized all the information and saved the data in hard drives. The details about the dataset are following.

### 3.2. Subjects and Activities

During the collection of data, 17 young healthy subjects without any impairment (9 male and 8 female) ranging from 18–24 years old, mean height of 1.66 m and mean weight of 66.8 kg, were invited to perform 11 different activities. Table 4 summarizes the statistics of the subjects.

The activities performed are related to six simple human daily activities (walking, standing, picking up an object, sitting, jumping and laying) and five human falls (falling forward using hands, falling forward using knees, falling backwards, falling sitting in an empty chair and falling sideward). These types of activities and falls were chosen from the analysis of those reported in literature [4,29]. Falls occurs performing a great variety of circumstances and manners [30]. We tried to simulate circumstances of falls when tripping, sitting and in different directions. We selected the most commonly ADL and in particular, picking up an object was included given that it is common to mistake this activity with a fall. All daily activities were performed during 60 s, except jumping that was performed during 30 s and picking up an object which it is an action done once within a 10-s period. A single fall was performed in each of the three ten seconds period trials. Time windows for daily activities were selected to cover them in at least the duration time reported in similar studies [13,14,28]; while window time for falls was selected based on the 6-s safe period after fall occurrence, as reported in [13]. For all these activities, a mattress was located in the falling area to prevent injuries. Each activity was performed three times (trials) by each young healthy subject without any impairment. Table 5 summarizes the activities and the duration each trial takes in the final dataset.

### 3.3. Ethical Approval and Consent to Participate

The Research Committee of Engineering Faculty of Universidad Panamericana approved all the study procedures. All healthy young adults without impairment that participated in this study previously filled out an agreement with the principal investigator and the Faculty of Engineering, considering the regulations and data policies applicable. The decision to participate in these experiments was voluntary.

### 3.4. Sensors and Distribution

In order to collect data from young healthy subjects without any impairment, we consider a multimodal approach for sensing the activities in three different ways using wearables, context-aware sensors and cameras, all at the same time. We used a controlled laboratory room in which light intensity does not vary, and the context-aware and cameras remain in the same position during the data collection process. However, we decided to maintain the windows visible, thus in some cases there are recordings from cameras that show people moving in the background.

We use five Mbientlab MetaSensor wearable sensors collecting raw data from the 3-axis accelerometer, the 3-axis gyroscope and the ambient light value. These wearables were located in the left wrist, under the neck, at right pocket of pants, at the middle of waist (in the belt), and in the left ankle. Also, one electroencephalograph (EEG) NeuroSky MindWave headset was occupied to measure the raw brainwave signal from its unique EEG channel sensor located at the forehead. The sensor position has always been a challenge in fall detection and human activity recognition. According to [4,16], waist, thigh (pocket), wrist, chest, foot are the preferred locations for accelerometers and accelerometers embedded in smart devices. We chose to position one IMU in the left wrist simulating that the participant is wearing a smart watch. We placed another IMU in the right pocket simulating the place for wearing a smart phone. The sensor positions were chosen considering a right-handed person. A dominant versus non-dominant side position analysis is out of the scope of this work. The dominant side of the subjects is shown in Table 4.

**Figure 1:**
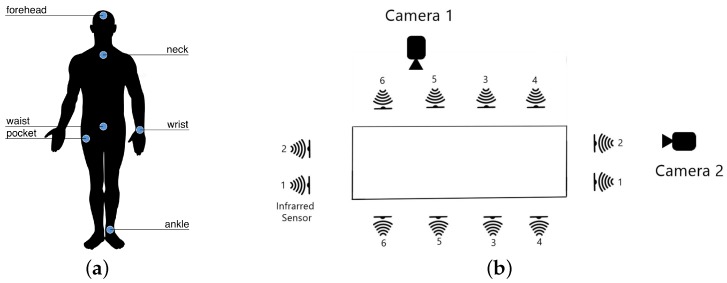
Distribution of the sensors. (**a**) Wearable sensors and EEG headset located at the human body. (**b**) Layout of the context-aware sensors and camera views.

**Figure 2:**
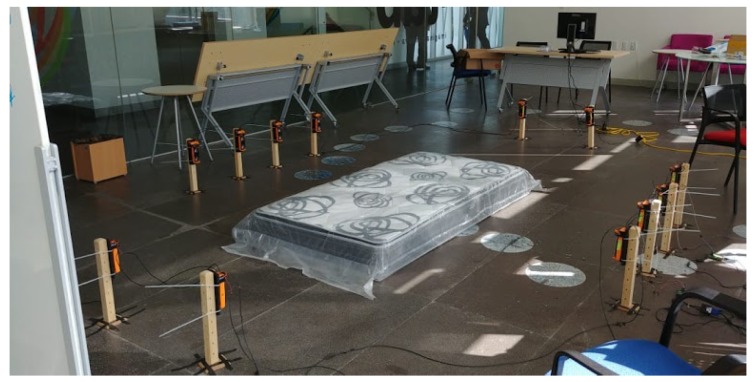
Implementation of the laboratory room for data collection.

As context-aware sensors, we installed six infrared sensors as a grid 0.40 m above the floor of the room, to measure the changes in interruption of the optical devices, where 0 means interruption and 1 no interruption. Lastly, two Microsoft LifeCam Cinema cameras were located at 1.82 m above the floor, one for a lateral view and the other for a frontal view. Figure 1a shows the location of the wearables in the body and Figure 1b shows the layout of the context-aware sensors and cameras. A real photography of the laboratory with the devices is shown in Figure 2. In addition, Table 6 summarizes all the sensors occupied and the units of measurement for each channel.

### 3.5. Hardware Implementation, Data Pre-Processing and Consolidation

To gather all raw sensor signals, a local system was implemented. In this regard, two computers and three Raspberry Pi V3 were used as units of information. The wearable sensors and the EEG headset were connected directly to the two computers via Bluetooth (three wearable sensors to one computer, and two wearable sensors plus the EEG headset to another computer). In addition, each camera was plugged into each computer via USB cable. Additionally, the infrared sensors were connected in pairs to the Raspberry Pi modules. Before each subject started to perform the activities, all the sensors and cameras started to gather the data. Later on, these devices stopped collecting data much later than the ending of performance by the subject. All the data were saved as CSV-files in the different units of information, containing the timestamp and the raw values associated with each sensor. It is important to highlight that previously all units of information were set with the same time.

For consolidation purposes, all the data were pre-processed. Since the devices ran at different sampling rates, we decided to homogenize the sampling rate in the consolidated dataset. In that sense, we chose the camera with the fewest frames acquired (18 fps approx.), taking its time-stamps as reference. Then, only raw values at these time-stamps were included in the consolidated dataset. Only infrared sensors were too slow (4 Hz) for that sampling rate, thus upsampling was conducted using drop-sampling interpolation [31]. This upsampling procedure consists of repeating the last sampled value *n*-times (i.e., n=4 for our dataset) until the next sampled value is acquired. This upsampling data represents 10.3% of samples associated with infrared sensors. For data alignment, we recorded the starting and ending time-stamps of trials, and then we extracted information from devices only in this interval of time. That process was possible since all devices recorded time-stamps locally (previously calibrated), thus no data wrapping were required. Further details on pre-processing and consolidation processes of our dataset can be found in [32].

The final consolidated dataset contains 296,364 samples of raw sensor signals and images. These samples were collected at ∼18.4 Hz, and saved in around 812 GB of digital information.

### 3.6. Data Storage and Publishing

The UP-Fall Detection dataset comprises 11 activities, with three repetitions each, performed by 17 young healthy subjects without any impairment. All activities were measured using 14 devices and 44 multimodal sensor signals. This dataset aims to cover different human falls and simple activities for further analysis, benchmarck and design of fall detection and/or HAR systems. After pre-processing, the public UP-Fall Detection dataset considers two main components: (a) the consolidated dataset, and (b) the feature dataset. It is remarkable to say that the dataset has missing values, and these are reported in Table 7.

#### 3.6.1. Consolidated Dataset

This is the core dataset. It comprises clean and synchronized information of the activities performed by 17 young healthy subjects without any impairment. Due to formatting, the dataset is separated in data from sensors and images from cameras.

**Figure 3:**
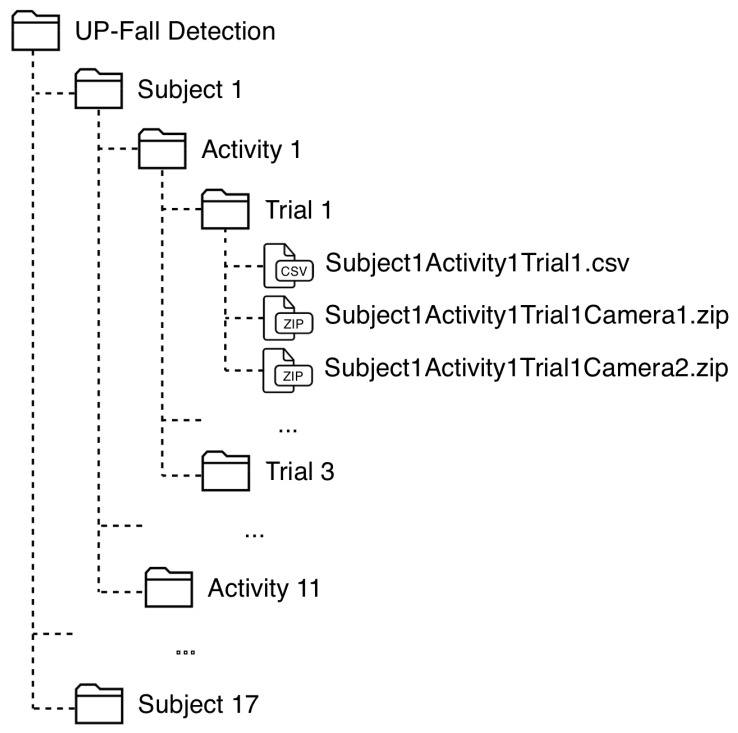
Organization of the consolidated dataset.

The data is organized into CSV-files (data from sensors) and ZIP-files (images from cameras) as follows. There are 17 folders, one per subject. Inside each folder, there are 11 sub-folder, one per activity. At the inside of these sub-folders, there are other three sub-folders, one per trial. In each sub-folder, there is one CSV-file containing the pre-processed sensor signals of that attempt and two ZIP-files containing the images recorded of that attempt for the both cameras, one file per camera. Figure 3 shows the organization of this dataset.

The name of each CSV-file is written as: SubjectXActivityYTrialZ, where X is the Subject ID, Y is the Activity ID and Z is the number of trial (1−3); and the ZIP-files are named as: SubjectXActivityYTrialZCameraW, where W is the number of camera (1 or 2).

Each CSV-file contains samples with: a column with the timestamp, 42 columns related to the sensor signals, and three columns with the number of subject, activity and trial. Table 8 shows the organization of this CSV-file related to the number of columns. It is important to highlight that falls consider three states in the activity: standing, falling and laying; while picking up an object considers also three states: standing, picking up and standing. In those cases, the values in the activity column of the files changes depending on the state. It is important to highlight that daily activity labels were tagged automatically using the time-stamps, while fall trials were tagged manually by inspection on the camera views. Just one expert tagged the samples and one person revised this task.

**Figure 4:**
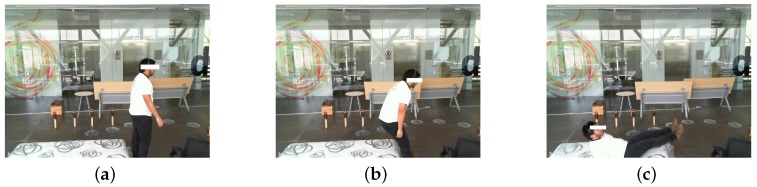
Examples of images inside a ZIP-file.

On the other hand, each ZIP-file contains a set of RGB images in PNG format. These images have a file name exactly as the timestamp when they were taken, so that they can be related easily with the data from sensors. Figure 4 shows a set of images that are collected in the dataset. It is important to notice that although the dataset was collected from falls simulated by young healthy subjects without any impairment, we incorporated non-fall activities and is highly imbalanced as suggested in [33] in order to simulate sporadic falls of real-world conditions.

#### 3.6.2. Feature Dataset

In most fall detection or HAR systems, feature extraction is part of the workflow. In this regard, we decided to extract features from the consolidated dataset. For this purpose, we did three different feature datasets depending on the window size: (a) one-second, (b) two-second and (c) three-second. All the feature datasets consider 50% of overlapping. Due to formatting, all these datasets are separated in features from sensors and image features from cameras.

**Figure 5:**
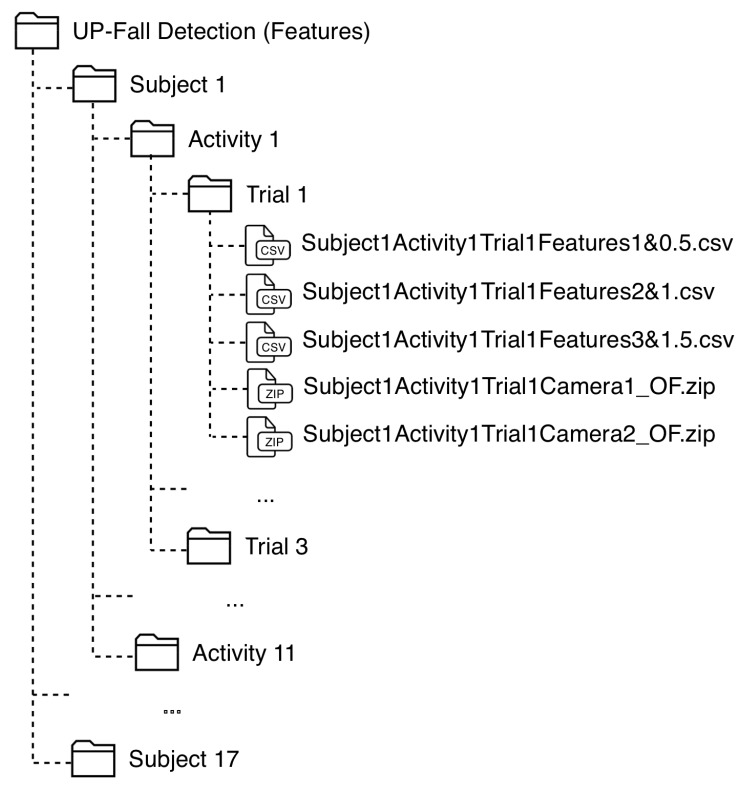
Organization of the feature dataset.

The data is organized into CSV-files (features from sensors and divdided by window size) and ZIP-files (image features from cameras) in the same way as in the consolidated datasets: 17-folders (subjects), each one with 11 sub-folders (activities), with three sub-folders (trials) each. At each sub-folder, there are three CSV-files containing the features extracted for each sensor signal of that trial, with three window sizes, and two ZIP-files containing the image features extracted from the image sequences at that attempt for the both cameras, one file per camera. Figure 5 shows the organization of this dataset.

The name of each CSV-file is written as: SubjectXActivityYTrialZFeaturesP&Q, where X is the Subject ID, Y is the Activity ID, Z is the number of trial (1–3), P is the window size (1–3 s), and Q is the size of the overlapping (0.5, 1 and 1.5 s). The ZIP-files are named as: SubjectXActivityYTrialZCameraW_OF, where W is the number of camera (1 or 2).

Each CSV-file contains window samples, each one with: a column with the timestamp at the beginning of the window, 756 columns related to 18 features extracted for each of the 42 sensor signals, and three columns with the number of subject, activity and trial. Table 9 shows the organization of these CSV-files. Activities such as falls and picking up an object were considered in the same way as in the consolidated dataset. In addition, the value reported at the activity column was calculated as the most frequent activity value over the entire window. To this end, the 18 features extracted are summarized in Table 10 for temporal features and in Table 11 for frequency features.

**Figure 6:**
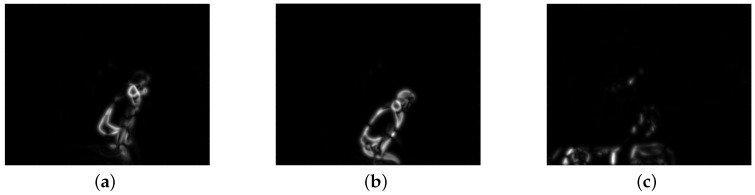
Examples of image features from cameras inside a ZIP-file.

One the other hand, each ZIP-file contains a set of compressed CSV-files that represents the relative displacement of pixels in two consecutive images, computed by an optical flow method. The latter approach is a methodology that allows calculating the apparent displacements of objects in an image sequence, these displacements, in general, are associated with brightness variations and can give correspondence information between the pixels of consecutive images [41]. For this dataset, the Horn and Schunck optical flow method was computed [42]. Figure 6 shows a set of images that can be interpreted from the information collected in the feature dataset. It is important to highlight that feature extraction over the images are not windowed.

## 4. Use Cases

In order to present examples of use cases in which our dataset can be useful, we propose two use case scenarios: (i) modalities configuration and (ii) a benchmark of machine learning models. For each use case, different goals were proposed.Seven experiments were designed to achieve these goals:

- *Experiment 1 (IR).* Fall detection using data only from infrared sensors.
- *Experiment 2 (IMU).* Fall detection using data only from wearable IMUs.
- *Experiment 3 (IMU+EEG).* Fall detection using data from all wearable IMUs and the EEG headset.
- *Experiment 4 (IR+IMU+EEG).* Fall detection using data from all infrared sensors, all wearable IMUs and the EEG headset.
- *Experiment 5 (CAM).* Fall detection using only data from cameras.
- *Experiment 6 (IR+CAM).* Fall detection using data from all infrared sensors and cameras.
- *Experiment 7 (IMU+EEG+CAM).* Fall detection using data from all wearable IMUs, EEG headset and cameras.

The above combinations are not exhaustive. Experiments with all sort of combinations using only some sensors with different locations and/or modalities can be designed depending of the purpose of the experiments. Different and new algorithms can also be used.

### 4.1. Case 1: Modalities Configuration

Given the availability and affordability of wearable sensors, ambient and vision sensors and devices, it is more common to use different modalities for fall detection. Nevertheless, as discussed before, it is important to choose the right combination of modalities and location of sensors. In this case scenario, we exemplify how a comparative analysis can be done for the purpose of selecting the combination of sensors and devices with the best predictive capability.

### 4.2. Case 2: Benchmark of Machine Learning Models

Another important use of our dataset is the possibility to fairly compare different algorithms, systems and configurations. In this case scenario, we propose a comparative analysis of different machine learning algorithms. In this example we compare the performance of four well-known methods typically use in fall detection and human activity recognition systems [9,13,27,28,34,43]:

- *Random Forest (RF).* This is an ensemble method made of decision trees, in which an input is processed through the forest of decision trees and computes the output class as the mode of the response class given by the trees. This technique is employed in many fall detection and activity recognition systems [43].
- *Support Vector Machine (SVM).* This method maps the inputs to a different space in which a hyper-plane, optimized by training, separates the output classes. It occupies a kernel for suitable hyper-plane separation. It is a very popular classification method in fall detection systems [9].
- *Multi-Layer Perceptron (MLP).* This is a neural network with perceptron (i.e., threshold activation function) units, employed as a general nonlinear classification [44].
- *k-Nearest Neighbors (kNN).* This is an instance-based method that compares an input with the *k*-nearest neighbor training points and determines the output response based on the most frequent class observed in the *k* neighbors [44].

## 5. Experiments and Results

**Figure 7:**
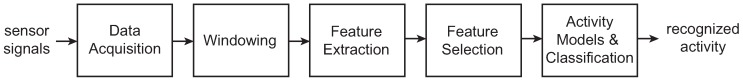
Activity recognition chain methodology adopted in the fall detection system.

We adopted the activity recognition chain (ARC) approach [34] to develop the workflow of the fall detection system aiming to test the case scenarios described below. This methodology considers five main steps: (i) data acquisition, (ii) windowing, (iii) feature extraction, (iv) feature selection and (v) activity models and classification. A detailed description of each step are presented following. Figure 7 shows the ARC methodology adopted for the experiments.

### 5.1. Data Acquisition

The first step of the ARC approach is to acquire data from the sources. This was already explained in Section 3.6.1. In summary, we collected data from 14 sources (e.g., wearables, ambient sensors, cameras) all connected to two computers that stored the information locally. These data were consolidated in a clean and synchronized dataset of the 11 activities, three attempts each, performed by 17 young healthy subjects without any impairment.

### 5.2. Windowing and Feature Extraction

The second step in the methodology considers to divide the raw signals in windows in order to extract relevant features, as described in Section 3.6.2. We tested the fall detection system using three different window sizes: (a) one-second, (b) two-second and (c) three-second. An overlapping of 50% were considered in all the cases. Then, at each window, we extracted 12 temporal and six frequency features (see Table 10 and Table 11). Windowing and feature extraction processes are fully described in Section 3.6.2.

For images, feature extraction was computed as follows. First, for each camera, we retrieved all image features inside a window. These features are the horizontal and vertical relative movements in the scenes, known as *u* and *v* respectively. These *u* and *v* components are two numeric matrices with the same size of the original images. For interpretability, we combined these two components resulting in the magnitude of the relative movement as shown in (1), where *d* is the resultant matrix of size equals to the original image.

(1) $$d_{i,j}=\sqrt{u_{i,j}^{2}+v_{i,j}^{2}}$$

To minimize computational effort in following steps, we resized the resultant matrix *d* from 640×480 to 20×20 size. After that, we reshaped matrix *d* in a row vector of 400 elements. Lastly, all these row vectors from image features inside a window were averaged. Thus, a 400-row vector was obtained for each window, representing the features for images.

To this end, we obtained 756 features from sensors (wearables and ambient ones) and 800 features from the two cameras, getting 1556 features in total for each window size setting.

### 5.3. Feature Selection

The third step of the ARC methodology is to select a subset of features in order to reduce the dimensionality and simplify the development of the models. Feature selection was applied to each consolidated dataset resulting from the process of feature extraction described in Section 5.2. For each of the seven experiments described in the use cases (see Section 4), feature selection was done using the following techniques: (i) a scheme-independent attribute subset evaluator using correlation-based feature selection (Weka.CfsSubsetEval), and (ii) three ranker methods based on attribute correlation, attribute relief and attribute classification (Weka.CorrelationAttributeEval, Weka.ReliefAttributeEval and Weka.ClassifierAttribute) [45].

In [45], Witten and Frank state that there are attribute selection is normally done with two methods: searching the space of attribute subsets and evaluating each one or evaluating the attributes individually, sort them and discarding attributes that fall below a cutoff point. We combined these two methods using one attribute subset evaluator method and three ranker methods for feature selection. The scheme-independent technique considers the individual predictive ability of each feature and the degree of redundancy of a given subset of features. Two search methods were considered inside this technique: best-first and greedy step-wise. In terms of the rankers, the first one evaluates the worth of a feature measuring Pearson’s correlation between the given attribute and the class, the second one alleviates the evaluation of correlation from attributes, and the third one use classification for select the most appropriate attributes. In the latter, two classifiers were proven for attribute ranking: ZeroR and Decision Table.

In summary, the most relevant attributes were selected for each case. The following steps were used for feature selection:

1. Revise features with missing values and select those features with consistent information.
2. Evaluate the worth of each attribute using the five techniques described above.
3. Select subsets of one hundred of the best attributes determined by each of five feature selection methods.
4. Calculate the frequency of appearance of each feature in all the selected subsets.
5. If a feature appears more than one time in these subsets, the feature was selected.
6. Sort features according to frequency of appearance.
7. Perform an incremental analysis of predictive power of features using Random Forest classification and accuracy metric.
8. Select a subset of the most relevant features for subsequent classification.

**Figure 8:**
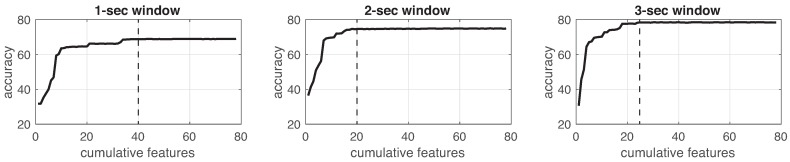
Accuracy performance on training using cumulative features for *Experiment 1*: IR. From left to right, it presents the performance using: 1-second length windowing, 2-second length windowing and 3-second length windowing. The dash line reports the number of cumulative features employed for the next steps.

**Figure 9:**
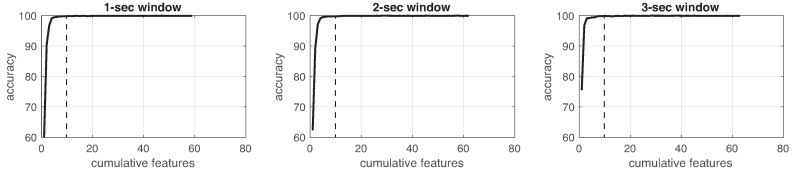
Accuracy performance on training using cumulative features for *Experiment 2*: IMU. From left to right, it presents the performance using: 1-second length windowing, 2-second length windowing and 3-second length windowing. The dash line reports the number of cumulative features employed for the next steps.

**Figure 10:**
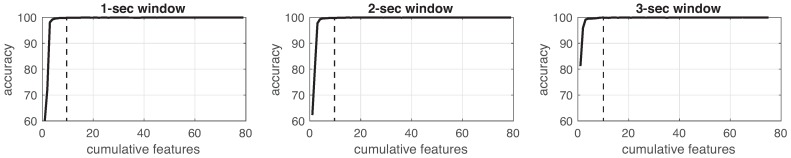
Accuracy performance on training using cumulative features for *Experiment 3*: IMU + EEG. From left to right, it presents the performance using: 1-second length windowing, 2-second length windowing and 3-second length windowing. The dash line reports the number of cumulative features employed for the next steps.

**Figure 11:**
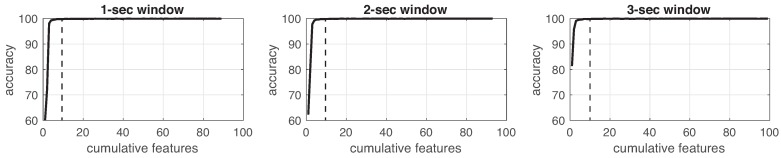
Accuracy performance on training using cumulative features for *Experiment 4*: IR + IMU + EEG. From left to right, it presents the performance using: 1-second length windowing, 2-second length windowing and 3-second length windowing. The dash line reports the number of cumulative features employed for the next steps.

**Figure 12:**
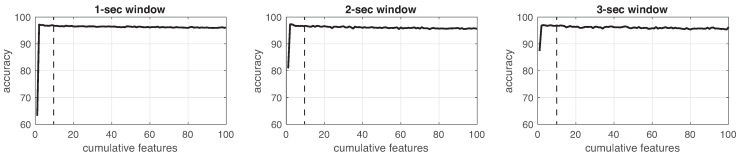
Accuracy performance on training using cumulative features for *Experiment 5*: CAM. From left to right, it presents the performance using: 1-second length windowing, 2-second length windowing and 3-second length windowing. The dash line reports the number of cumulative features employed for the next steps.

**Figure 13:**
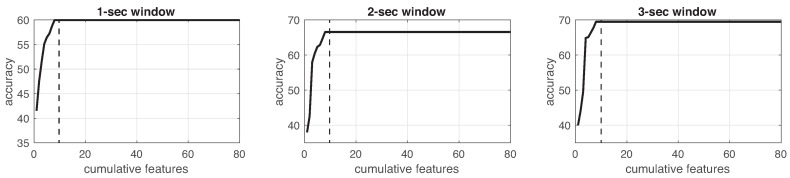
Accuracy performance on training using cumulative features for *Experiment 6*: IR + CAM. From left to right, it presents the performance using: 1-second length windowing, 2-second length windowing and 3-second length windowing. The dash line reports the number of cumulative features employed for the next steps.

**Figure 14:**
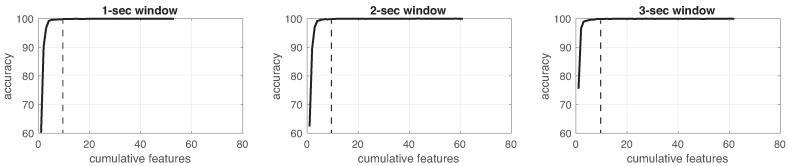
Accuracy performance on training using cumulative features for *Experiment 7*: IMU + EEG + CAM. From left to right, it presents the performance using: 1-second length windowing, 2-second length windowing and 3-second length windowing. The dash line reports the number of cumulative features employed for the next steps.

This process was implemented for each experiment in the three different window sizes. Figure 8, Figure 9, Figure 10, Figure 11, Figure 12, Figure 13 and Figure 14 show the incremental analysis of predictive power of features in terms of the accuracy. From left to right, each graph shows the accuracy obtained when using 1-s, 2-s and 3-s windowing. In addition, each vertical dashed line represents the number of features finally selected for building the machine learning models, as reported below.

### 5.4. Activity Models and Classification

Building machine learning models for classification is the next step in the workflow. In this work, four classification methods were applied to each subset of features extracted in each experiment. Table 12 summarizes the parameter settings of these models. Experiments were performed using 70% of the dataset for training and 30% for testing. Ten rounds of cross-validation were performed using different random partitions done by samples over each of the selected classification methods. In machine learning literature [46,47], it is suggested the determination of *k*-fold configuration as follows: (i) the value of *k* is chosen such as each trained group of data is large enough to be statistically representative and typically is performed with exhaustive experimentation [46], or (ii) if not exhaustive experimentation is done, the most common *k* chosen is 5- or 10-folds as these values have shown empirically to yield test error rate estimates that suffer neither from excessively high bias nor from high variance [47]. In addition, related works in fall detection commonly report using 10-fold configuration although it is difficult to compare works given the great variety of datasets, classification tasks, prediction techniques and evaluation metrics (c.f. [12,13,15,28]). Thus, we considered that exhaustive approach is computational expensive and was not necessary as we are only presenting an example of use of our dataset; we therefore decided to choose a 10-fold configuration based on the related work and the common practices reported in machine learning [46,47]. It is important to notice that for each size of window, experiments with seven combinations of modalities were performed using four classification methods.

For the experiments, we measure the performance of the classification models using five metrics [48]: *accuracy*, *precision*, *sensitivity*, *specificity* and *F${}_{1}$-score*, as shown in (2)–(6); where TP and TN are the true positives and true negatives, and FP and FN are the false positives and false negatives.

(2) $$accuracy=\frac{TP+TN}{TP+TN+FP+FN}$$

(3) $$precision=\frac{TP}{TP+FP}$$

(4) $$sensitivity=\frac{TP}{TP+FN}$$

(5) $$specificity=\frac{TN}{TN+FP}$$

(6) $$F_{1}-score=2\cdot \frac{precision\times sensitivity}{precision+sensitivity}$$

### 5.5. Results

After completing the ARC workflow for all the experiments, we obtained the performance evaluation of each multimodal approach in the different window sizes, as summarized in Table 13. It reports the best performance based on the F${}_{1}$-score (and in parenthesis the machine learning model that produces the best result) obtained from the combination modality-window size. The mean of the ten fold-cross validation for each method was compared, and the best result is reported in addition with its standard deviation. This was done for each of the different experiments.

**Figure 15:**
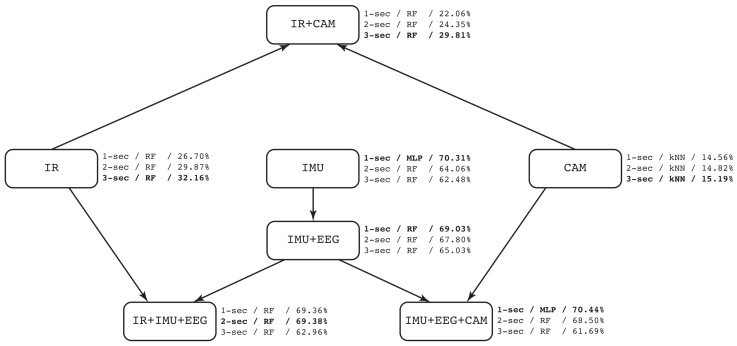
Graphical description of the different modalities. Information comprises: window size/best ML-model/mean F${}_{1}$-score. Bold text represents the best performance.

The following analysis is based on F${}_{1}$-score. As shown in Table 13, simple modalities for IR and CAM got bad performance, 32.16% and 15.19% respectively, except with IMUs-only sensors that reached 70.31%. When combining simple modes, such as IR + CAM, results were not better (29.81%). Following IMU + EEG, it obtained slightly less results (69.03%) than IMUs-only. However, adding more devices in modalities promotes better results. It can be seen at IR + IMU + EEG that reached 69.38% in contrast to IR-only (32.16%) or IMU + EEG (69.03%). In the same way, IMU + EEG + CAM obtained 70.44% in comparison with CAM-only (15.19%) or IMU+EEG (69.03%). Figure 15 shows a graphical representation of the different modalities and the performance (mean F${}_{1}$-score), already discussed above. To this end, *Case 1* shows that having multimodal devices using IMUs and EEG headset wearables in combination with cameras, the performance is better than using only one type of devices. This validates that multimodal approach has better predictive capability than the other combinations considered.

**Figure 16:**
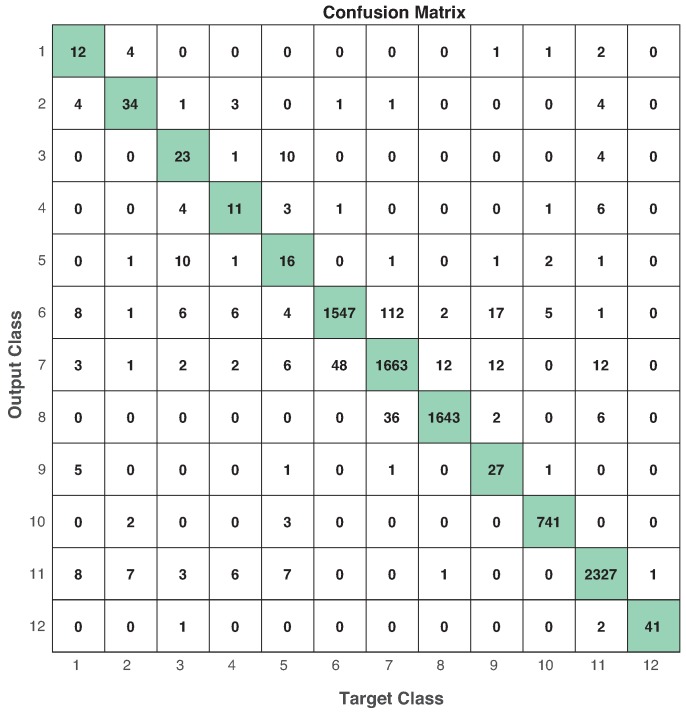
Confusion matrix in testing using MLP with 1-second window size in IMU + EEG + CAM. Numbers in diagonal represent the times a target class is estimated correctly. Performance: accuracy=95.0%, precision=77.7%, sensitivity=69.9%, specificity=99.5% and $F_{1}-score=72.8\%$.

On the other hand, we obtained the performance evaluation of each modality in the different machine learning models. Table 14 shows the best performance based on F${}_{1}$-score (and in parenthesis the window size that produces the best result) obtained from the combination modality-model. These results correspond to *Case 2* on benchmark of machine learning models. In terms of the ML models, RF seems to be the best predictive model in the whole experiment. However, we can identify that RF and MLP are the two related classifiers to multimodal approach (see Figure 15). From Table 14, IR+IMU+EEG reached a performance of 69.38% (RF) and 68.19% (MLP), in contrast to 53.94% (SVM) and 60.36% (kNN). The same behavior is shown in IMU+EEG+CAM with the highest performance got from RF (69.36%) and MLP (70.44%). In CAM, kNN was the most useful among the others, and we consider this happened because vision features were selected to be pixels representing the relative motion between frames. In that sense, an instance-based ML model would be better in this case than the others. Surprisingly, SVM was not be selected in any well-performed combination. Thus, this experiment shows the usefulness of having different modalities to fairly compare ML-models in the same circumstances. To this end, Figure 16 shows the confusion matrix of the best ML-model found using IMU + EEG + CAM modality with MLP and 1-second window size.

### 5.6. CNN for Vision

As shown before, ML-models cannot predict falls and activities when using vision features only (CAM). Thus, we conducted a small experiment with convolutional neural networks (CNN) to determine the feasibility of our database to predict falls/activities using only vision.

For this experiment, we use a CNN adapted for our raw video recordings. CNN is a type of deep learning neural network inspired on the biological process of connectivity pattern in neurons of animal visual cortex. CNN have shown to be versatile in automatic feature extraction procedures, using a suitable amount of samples in training phase. For instance, Núñez-Marcos et al. [49] showed that CNN with optical flow can lead in fall detection systems.

**Figure 17:**
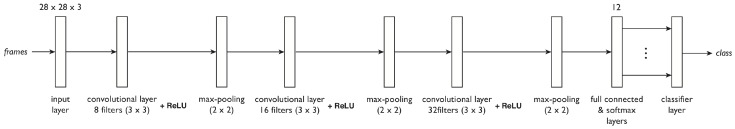
CNN topology using raw video recordings.

In our experiment, the proposed CNN receives as input a frame from the video recordings and estimates the fall/activity performed by the present subject. Figure 17 shows the architecture of the employed CNN with the following layers: a convolutional layer with 8 filters of size 3×3 with a rectified linear unit (ReLU) and a max-pooling of size 2×2 layers; then, a convolutional layer with 16 filters of size 3×3 with a ReLU and a max-pooling of size 2×2 layers; after that, a convolutional layer with 32 filters of size 3×3 with a ReLU and a max-pooling of size 2×2 layers; and, finally, there is a fully-connected layer with output size 12 and soft-max function. We trained the CNN using the stochastic gradient descent algorithm with initial learning rate of 0.001, regularization coefficient 0.004, maximum number of epochs 5, and mini-batch size of 100.

**Figure 18:**
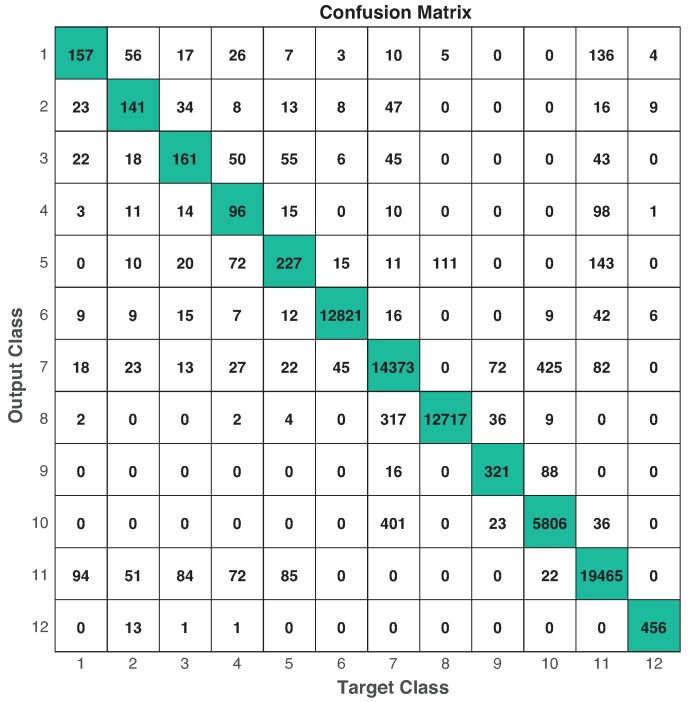
Confusion matrix in testing using video recordings. Numbers in diagonal represent the times a target class is estimated correctly. Performance: accuracy=95.1%, precision=71.8%, sensitivity=71.3%, specificity=99.5% and $F_{1}-score=71.2\%$.

The training data for CNN consisted on 140,451 samples and the testing data on 70,145 samples. Only camera 1 was used for training and testing, and images were re-sized to 28×28 pixels. We ran 5-fold cross-validation for training process, based on the procedure reported in [49] and the common practices considered in machine learning [46,47], and we selected the best CNN classifier using the accuracy metric over the training set. After that, we validated our CNN over the testing data, performing: accuracy=95.1%, precision=71.8%, sensitivity=71.3%, specificity=99.5% and $F_{1}-score=71.2\%$. This is also shown in the confusion matrix depicted in Figure 18. In this case, class 12 represents an *unknown* activity.

The above CNN demonstrated that our video recordings can be used for falls/activities detection. In addition, it is important to consider the combinations of ML-models and features for classification.

## 6. Discussion

To the best of our knowledge, there are limited multimodal datasets with different human activities including falls that are publicly available, as shown in Table 3. On the other hand, there is a need for new multimodal datasets to fairly compare fall detection solutions. It is also important for research communities to assess new machine learning algorithms. Our proposed multimodal UP-Fall Detection Dataset provides a useful resource for conducting experiments with various goals. With this in mind, we presented two use cases whose results are discussed below.

Regarding the first use case of modalities configuration, we can observe that results (Table 13) are better when IMUs were included [10]. Although new sensors and modalities are being used in related work, accelerometer is proven to be a good choice for fall detection. Comparing IMU + EEG and IR + IMU + EEG, we can observe that although IMUs have the most predictive power regarding fall detection, ambient sensors in this case infrared sensors, contribute to slightly improve the classification results. With respect to CAM modality in which only cameras were considered, results show poor fall detection with the selected features and classifiers. Results are improved when IMUs are combined with cameras as expected (IMU + EEG + CAM). Furthermore, our experiment with CNN using raw video recording shows that this approach highly improves the performance of fall detection.

From the benchmark of machine learning models experiments shown in Table 14, we can observe that RF algorithm presents the best results in almost all experiments. These results can be bias given that feature selection was assessed with RF model. The performance of MLP and SVM are not very consistent as seen in Figure 15 and Table 14. In addition, standard deviation of these techniques shows more variability. Surprisingly, SVM did not perform in the top of ML-models tested in this work.

On one hand, in neither the experiments, different window lengths represent significant improvement among the others. As observed, the 2-s window size less supports the performance of the classifiers. For instance, looking at Table 13 or Figure 15, 1-s window length promotes better performance in devices with more information, e.g., IMU (5 devices with 7 channels); while 3-s window size supports better performance in devices with less information, e.g., IR (6 devices, 1 channel). On the other hand, the sampling rate (18 Hz) of the consolidated dataset can confirmed to be useful since classification reported well performance. This sampling rate was obtained by a trade-off between the highest frequency rate of devices (IMUs) and the lowest one (infrared sensor). Even though this sampling rate is not high enough, literature reports that having a larger sampling rate values does not improve the performance of classifier methods [9].

With regards to performance, it is very difficult to compare our results with the ones reported in literature. First of all, the machine learning task is not always the same. Some related works use a class that only specifies fall/not fall and other works try to classify each of the different activities or types of fall. The latter is the machine learning task performed in our approach. The difference in types of data and types of evaluation metric used is also very diverse as we can see in Table 1, Table 2 and Table 3. It can be said that in general terms, our results are competitive with respect to the reported works.

It is important to consider improvements to machine learning strategies, so falls and activities detection can be improved significantly due to the results observed, for instance, in Figure 16 and Figure 18. For example, hierarchical classification, deep learning and transfer learning approaches would be adopted. To this end, other experiments in multimedia and human activity recognition use cases could be designed in which our dataset will be valuable.

### Limitations

This study has some limitations. In data collection, all activities were performed in the same order and trials were performed consecutively. Falls were self-initiated and subjects fell onto a protective mattress that damped the impact of the simulation. This is a difference between real falls which generally occur towards hard materials and no intuitive reaction trying not to fall was recorded, limitation considered also in [50]. Also, the sensor positions were chosen considering a right-handed person. A dominant versus non-dominant side position analysis is out of the scope of this work, but dominant side of the subjects is shown in Table 4. It is important to notice that this dataset was thought for simple and non-overlapping activities, so down-sampling rates in IMUs (18 Hz) do not affect stationary fall predictions. This might be a limitation if the dataset would be used for real life predictions during dynamic situations (e.g., concurrent falls-and-activities).

In addition, falls were simulated by young healthy subjects without any impairment for safety reasons, nevertheless we are aware that some differences can be found with real falls in elderly people. We cannot guarantee that fall prediction for older or impaired adults can be done with a model build directly using our dataset. Hence, this dataset can be used for transfer learning experiments for prediction in elderly people or adults with impairments.

## 7. Conclusions

In this paper, we present a publicly available UP-Fall Detection Dataset to address the lack of multimodal datasets for human activity recognition and fall detection. Execution of activities was done by 17 healthy young subjects without any impairment. This dataset provides a wide range of experimental possibilities among multimedia, human activity recognition, and machine learning communities.

We aim to contribute particularly to motivate the research communities to develop various and robust fall detection systems that can reduce the consequences of falls. The dataset is a valuable experimental resource that can leverage the development of online detection technologies and physical devices for fall detection. We encourage the aforementioned communities to use our dataset.

We presented two use case scenarios to demonstrate examples of experimental possibilities: modalities configuration and benchmark of machine learning models. Another use case scenario could be identifying the best location and position of accelerometers and/or cameras. Our results demonstrated that fall detection models can be trained and tested with UP-Fall Detection Dataset.

For future work and as part of our on-going project, we are developing a multimodal fall detection system that can detect falls and emit an alert in real-time.

## Figures and Tables

**Table 1:** Wearable-based databases for fall detection.

Dataset	Type of Sensors	Position	Subjects	Fall Types	Other Activities	Trials	Method	Performance
DLR Dataset [11]	one IMU accelerometer	belt	16 (23 to 50 years old)	falling	walking, running, standing, sitting, laying, jumping		Bayesian techniques	recall 100% precision 80%
MobiFall Dataset [12]	smartphone accelerometer and gyroscope sensors	trouser pocket	24 (22 to 47 years old)	fall forward from standing, use of hands to dampen fall; fall forward from standing, first impact on knees; fall sidewards from standing, bending legs; fall backward while trying to sit on a chair	standing, walking, jogging, jumping, stairs up, stairs down, sit chair, car-step in, car-step out	3 to 6	*k*-nearest neighbor	accuracy fall detection 99.12%, fall classification 83.06%
tFall [13]	two smartphones accelerometers	worn in two pockets (left and right)	10 (20 to 42 years old)	eight types of fall: fall forward, fall backward, fall left and right-lateral, syncope, sitting on empty chair, falls using compensation to prevent the impact	ADL	3	neural networks, support vector machines	auc 95.29%, sensitivity 90.75%, specificity 89.65%
Vilarinho et al. [14]	smartphone and smartwatch	thigh pocket and wrist	3	12 types of fall	7 ADL		threshold	accuracy 68%, sensitivity 63%, specificity 78%
UMAFall [16]	smartphone and four wearable sensors	thigh pocket and ankle, waist, right wrist and chest	17 (18 to 55 years old)	3 types of fall, backwards, forwards, lateral	8 ADL	3	threshold	
SisFall [15]	self developed device with two accelerometeres and one gyroscope	waist worn	23 young and 15 elderly adults	15 types of fall	19 ADL	1 or 5	threshold	young: accuracy 92.684%, sensitivity 95.74%, specificity 89.62% elderly: accuracy 88.112%, sensitivity 79.46%, specificity 96.76%

**Table 2:** Vision-based datasets for fall detection.

Dataset	Camera Type	Camera Viewpoints	Subjects	Fall Types	Other Activities	Trials	Variants	ML Method	Performance
SDUFall [20]	one Kinect	one	10	falls in different directions	falling down, bending, squatting, sitting, laying, walking	6 actions /10 times	carrying/not carrying, light on/off, position/direction changes		accuracy 79.91%, sensitivity 81.91% specificity 76.62%
EDF [21]	two Kinect	two	10	falls in eight different directions	picking up something, sitting on the floor, laying down, tying shoelaces, doing plank exercise	6 actions/20 times	different directions		
OCCU [21]	two Kinect	two	5	falls in eight different directions	picking up something, sitting on the floor, laying down, tying shoelaces, doing plank exercise	6 occluded falls; 5 actions/20 times	occluded falls		
Charfi et al. [22]	one Kinect	one		falls in different directions	walking, sitting down, standing up, crouching down, housekeeping, moving a chair) and falls (forward falls, falls when sitting-down, loss of balance)	NaN	four different locations (home, coffee room, office, lecture room) illumination variances and occlusions, cluttered and textured background	3D based real time fall detection SVM; eight inexpensive IP cameras	accuracy 99.6%, precision 94.2%, recall 98% specificity 99.6%
Mastorakis et al. [19]	one Kinect	one height of 204 cm	8	backward, forward, sideways	sitting, laying, picking up, sweeping, dusting	6 trials fall; 4 activities	slow activities imitate elderly person	human 3D bounding box	
Dovgan et al. [25]	six infrared cameras and infrared light sources		3	tripping, fainting, sliding from chair	walking, laying down, laying, sitting down, sitting	10	markers attached to ankles, knees, hips, shoulders, elbows and wrists; Health analysis	C4.5 and Support Vector Machine (SVM)	accuracy 95.7%
Auvinet et al. [23]	eight cameras	eight positions	1	forward fall, backwards fall, fall when sitting down, loss of balance (falling)	walking, standing up, laying, crouching, moving down, moving up, sitting, laying on a sofa, moving horizontally	NaN	occlusions, moving objects		

**Table 3:** Multimodal databases for fall detection.

Dataset	Type of Sensors	Camera Type/Position	Subjects	Fall Types	Other Activities	Trials	ML Method	Performance
UR [26]	one IMU with accelerometer	two Kinect	5	falling from standing position, falling down sitting on a chair	ADL	70 sequences		accuracy 94.99%, precision 89.57%, sensitivity 100%, specificity 91.25%
MHAD [27,28]	six accelerometers, four microphones	one motion capture system, four multi-view cameras arranged in four clusters, one Kinect system	12	falls were not considered	ADL	5 repetitions	support vector machines, *k*-nearest neighbors	accuracy 98.24%
Dovgan et al. [25]	Ubisense system and with an Xsens accelerometer	Ubisense tags attached to waist, chest and both ankles	10	tripping, fainting, sliding from chair	walking, laying down, laying, sitting down, sitting	2 falling, 3 ADL	C4.5 and Support Vector Machine (SVM)	accuracy 95.58% Ubisense; accuracy 57.96% accelerometer
UP-Fall Detection (our proposal)	five IMU, one EEG headset, six infrared in grid	two cameras/frontal and lateral	17	falling forward using hands, falling forward using knees, falling backwards, falling sitting in empty chair, falling sideward	walking, standing, sitting, picking up an object, jumping, laying	3 repetitions	random forest, support vector machines, neural networks, *k*-nearest neighbors	See resuts in Section 5

**Table 4:** Statistics of the subjects.

Subject ID	Age	Height (m)	Weight (kg)	Gender	Dominant-Side
1	18	1.70	99	Male	Right handed
2	20	1.70	58	Male	Right handed
3	19	1.57	54	Female	Left handed
4	20	1.62	71	Female	Right handed
5	21	1.71	69	Male	Right handed
6	22	1.62	68	Male	Right handed
7	24	1.74	70	Male	Right handed
8	23	1.75	88	Male	Right handed
9	23	1.68	70	Female	Right handed
10	19	1.69	63	Male	Right handed
11	20	1.65	73	Female	Right handed
12	19	1.60	53	Female	Right handed
13	20	1.64	55	Male	Right handed
14	19	1.70	73	Female	Right handed
15	21	1.57	56	Female	Right handed
16	20	1.70	62	Male	Right handed
17	20	1.66	54	Female	Right handed

**Table 5:** Activities performed by subjects.

Activity ID	Description	Duration (s)
1	Falling forward using hands	10
2	Falling forward using knees	10
3	Falling backwards	10
4	Falling sideward	10
5	Falling sitting in empty chair	10
6	Walking	60
7	Standing	60
8	Sitting	60
9	Picking up an object	10
10	Jumping	30
11	Laying	60

**Table 6:** List of devices for measurements.

Device ID	Device Name	Channel Name	Units	Sampling Rate	Signal ID
1	Wearable Ankle	X-Axis Accelerometer	g	100 Hz	1
		Y-Axis Accelerometer	g	100 Hz	2
		Z-Axis Accelerometer	g	100 Hz	3
		Roll Gyroscope	deg/s	100 Hz	4
		Pitch Gyroscope	deg/s	100 Hz	5
		Yaw Gyroscope	deg/s	100 Hz	6
		Luminosity	lux	50 Hz	7
2	Wearable Pocket	X-Axis Accelerometer	g	100 Hz	8
		Y-Axis Accelerometer	g	100 Hz	9
		Z-Axis Accelerometer	g	100 Hz	10
		Roll Gyroscope	deg/s	100 Hz	11
		Pitch Gyroscope	deg/s	100 Hz	12
		Yaw Gyroscope	deg/s	100 Hz	13
		Luminosity	lux	50 Hz	14
3	Wearable Waist	X-Axis Accelerometer	g	100 Hz	15
		Y-Axis Accelerometer	g	100 Hz	16
		Z-Axis Accelerometer	g	100 Hz	17
		Roll Gyroscope	deg/s	100 Hz	18
		Pitch Gyroscope	deg/s	100 Hz	19
		Yaw Gyroscope	deg/s	100 Hz	20
		Luminosity	lux	50 Hz	21
4	Wearable Neck	X-Axis Accelerometer	g	100 Hz	22
		Y-Axis Accelerometer	g	100 Hz	23
		Z-Axis Accelerometer	g	100 Hz	24
		Roll Gyroscope	deg/s	100 Hz	25
		Pitch Gyroscope	deg/s	100 Hz	26
		Yaw Gyroscope	deg/s	100 Hz	27
		Luminosity	lux	50 Hz	28
5	Wearable Wrist	X-Axis Accelerometer	g	100 Hz	29
		Y-Axis Accelerometer	g	100 Hz	30
		Z-Axis Accelerometer	g	100 Hz	31
		Roll Gyroscope	deg/s	100 Hz	32
		Pitch Gyroscope	deg/s	100 Hz	33
		Yaw Gyroscope	deg/s	100 Hz	34
		Luminosity	lux	50 Hz	35
6	EEG Headset	Raw Brainwave Signal	μV	512 Hz	36
7	Infrared 1	No Interruption	false(0)/true(1)	4 Hz	37
8	Infrared 2	No Interruption	false(0)/true(1)	4 Hz	38
9	Infrared 3	No Interruption	false(0)/true(1)	4 Hz	39
10	Infrared 4	No Interruption	false(0)/true(1)	4 Hz	40
11	Infrared 5	No Interruption	false(0)/true(1)	4 Hz	41
12	Infrared 6	No Interruption	false(0)/true(1)	4 Hz	42
13	Camera 1	Lateral View	640 × 480 px	18 Hz	43
14	Camera 2	Frontal View	640 × 480 px	18 Hz	44

**Table 7:** Missing values in the UP-Fall Detection dataset.

Subject ID	Activity ID	Trial	Device ID
2	5	all	6
5	all	all	2
6	10	2	14
8	11	2, 3	all
9	all	all	2

**Table 8:** Example of organization inside a CSV-file of the consolidated dataset. Each row represents one sample.

Timestamp	Sensor Signals	Subject	Activity	Trial
YYYY-MM-DDTHH:MM:SS.SSSSSS	data from Signal ID 1–42	Subject ID	Activity ID	no. trial
2018-07-04T12:04:17.734054	42-columns with numeric values	1	7	2
2018-07-04T11:22:48.920482	756-columns with numeric values	2	5	3
2018-07-04T15:49:23.302938	756-columns with numeric values	16	11	1

**Table 9:** Example of organization inside a CSV-file of the feature dataset. Each row represents one sample.

Timestamp	Features from Sensor Signals	Subject	Activity	Trial
YYYY-MM-DDTHH:MM:SS.SSSSSS	756 features from Signal ID 1–42	Subject ID	Activity ID	no. trial
2018-07-04T12:04:17.734054	756-columns with numeric values	1	7	2
2018-07-04T11:22:48.920482	756-columns with numeric values	2	5	3
2018-07-04T15:49:23.302938	756-columns with numeric values	16	11	1

**Table 10:** Temporal features extracted from all wearable and ambient devices of the consolidated dataset.

Features	References
mean	[34,35,36,37,38]
standard deviation	[36,37,39]
root mean square	[35]
maximal amplitude	[37,38]
minimal amplitude	[37,38]
median	[39,40]
number of zero-crossing	[35,37]
skewness	[38]
kurtosis	[34,38]
first quartile	[39,40]
third quartile	[39,40]
autocorrelation	[37,38]

**Table 11:** Frequency features extracted from the consolidated dataset.

Features	References
mean frequency	[35,37]
median frequency	[35]
entropy	[36,40]
energy	[34,36,40]
principal frequency	[38,39,40]
spectral centroid	[37,40]

**Table 12:** Parameter settings for ML-models of the benchmark.

ML-Model	Parameters
Random Forest	estimators =10
	min. samples split =2
	min. samples leaf =1
	bootstrap =true
Support Vector Machines	c=1.0
	kernel $=radial_{b}asis_{f}unction$
	kernel coefficient =1/features
	shrinking =true
	tolerance =0.001
Multi-Layer Perceptron	hidden layer size =100
	activation function =ReLU
	solver $=stochastic_{g}radient$
	penalty parameter =0.0001
	batch size =min(200,samples)
	initial learning rate =0.001
	shuffle =true
	tolerance =0.0001
	exponential decay (first moment) =0.9
	exponential decay (second moment) =0.999
	regularization coefficient $=1e^{-8}$
	max. epochs =10
*k*-Nearest Neighbors	neighbors =5
	leaf size =30
	metric =Euclidean

**Table 13:** The best performance (mean ± standard deviation) obtained for each modality, based on the F${}_{1}$-score, depending on the window size. The best model is written in parenthesis.

Modality	Window	Accuracy (%)	Precision (%)	Sensibility (%)	Specificity (%)	F${}_{1}$-Score (%)
IR	1 s (RF)	63.03±0.48	31.21±1.25	26.26±0.52	96.11±0.05	26.70±0.73
	2 s (RF)	65.51±0.69	32.95±2.43	29.15±1.22	96.42±0.07	29.87±1.47
	3 s (RF)	67.38±0.65	36.45±2.46	31.26±0.90	96.63±0.07	32.16±0.99
IMU	1 s (MLP)	95.49±0.26	73.05±1.90	69.40±1.47	99.56±0.02	70.31±1.48
	2 s (RF)	94.23±0.30	71.99±3.09	60.81±1.47	99.42±0.03	64.06±1.74
	3 s (RF)	95.39±0.45	69.36±3.61	60.29±2.10	99.54±0.05	62.48±2.33
IMU + EEG	1 s (RF)	95.93±0.30	74.15±1.29	66.29±1.67	99.60±0.03	69.03±1.48
	2 s (RF)	95.16±0.23	73.06±2.27	65.16±2.01	99.52±0.02	67.80±1.71
	3 s (RF)	95.60±0.35	72.39±2.92	62.34±2.76	99.56±0.03	65.03±2.31
IR + IMU + EEG	1 s (RF)	95.88±0.16	74.17±1.08	66.73±0.72	99.59±0.02	69.36±0.58
	2 s (RF)	95.12±0.37	74.64±1.65	66.71±1.99	99.51±0.04	69.38±1.72
	3 s (RF)	95.58±0.49	70.10±3.04	60.88±3.93	99.56±0.05	62.96±3.28
CAM	1 s (kNN)	32.51±0.42	14.40±0.65	15.06±0.49	92.93±0.04	14.56±0.54
	2 s (kNN)	34.01±0.69	14.65±0.70	15.30±0.52	93.10±0.07	14.82±0.60
	3 s (kNN)	34.03±1.12	15.33±0.73	15.54±0.57	93.09±0.12	15.19±0.53
IR + CAM	1 s (RF)	56.73±0.33	25.90±1.25	23.25±0.27	95.48±0.03	22.06±0.43
	2 s (RF)	60.00±0.44	27.90±1.44	25.86±0.68	95.84±0.05	24.35±0.83
	3 s (RF)	65.00±0.66	33.94±2.82	29.03±0.90	96.35±0.07	29.81±1.16
IMU + EEG + CAM	1 s (MLP)	94.32±0.31	76.79±1.59	67.30±1.42	99.43±0.03	70.44±1.25
	2 s (RF)	95.06±0.22	74.08±1.87	65.65±1.02	99.51±0.03	68.50±1.13
	3 s (RF)	95.19±0.22	70.33±3.09	58.69±1.81	99.52±0.02	61.69±1.97

**Table 14:** The best performance (mean ± standard deviation) obtained for each modality depending on the ML-model. The best window size is written in parenthesis.

Modality	Model	Accuracy (%)	Precision (%)	Sensibility (%)	Specificity (%)	F${}_{1}$-Score (%)
IR	RF (3 s)	67.38±0.65	36.45±2.46	31.26±0.89	96.63±0.07	32.16±0.99
	SVM (3 s)	65.16±0.90	26.77±0.58	25.16±0.29	96.31±0.09	23.89±0.41
	MLP (3 s)	65.69±0.89	28.19±3.56	26.40±0.71	96.41±0.08	25.13±1.09
	kNN (3 s)	61.79±1.47	30.04±1.44	27.55±0.97	96.05±0.16	27.89±1.13
IMU	RF (1 s)	95.76±0.18	70.78±1.53	66.91±1.28	99.59±0.02	68.35±1.25
	SVM (1 s)	93.32±0.23	66.16±3.33	58.82±1.53	99.32±0.02	60.00±1.34
	MLP (1 s)	95.48±0.25	73.04±1.89	69.39±1.47	99.56±0.02	70.31±1.48
	kNN (1 s)	94.90±0.18	69.05±1.63	64.28±1.57	99.50±0.02	66.03±1.52
IMU + EEG	RF (1 s)	95.92±0.29	74.14±1.29	66.29±1.66	99.59±0.03	69.03±1.48
	SVM (1 s)	90.77±0.36	62.51±3.34	52.46±1.19	99.03±0.03	53.91±1.16
	MLP (1 s)	93.33±0.55	74.10±1.61	65.32±1.15	99.32±0.05	68.13±1.16
	kNN (1 s)	92.12±0.31	66.86±1.32	58.30±1.20	98.89±0.05	60.56±1.02
IR + IMU + EEG	RF (2 s)	95.12±0.36	74.63±1.65	66.71±1.98	99.51±0.03	69.38±1.72
	SVM (1 s)	90.59±0.27	64.75±3.89	52.63±1.42	99.01±0.02	53.94±1.47
	MLP (1 s)	93.26±0.69	73.51±1.59	66.05±1.11	99.31±0.07	68.19±1.02
	kNN (1 s)	92.24±0.25	67.33±1.94	58.11±1.61	99.21±0.02	60.36±1.71
CAM	RF (3 s)	32.33±0.90	14.45±1.07	14.48±0.82	92.91±0.09	14.38±0.89
	SVM (2 s)	34.40±0.67	13.81±0.22	14.30±0.31	92.97±0.06	13.83±0.27
	MLP (3 s)	27.08±2.03	8.59±1.69	10.59±0.38	92.21±0.09	7.31±0.82
	kNN (3 s)	34.03±1.11	15.32±0.73	15.54±0.57	93.09±0.11	15.19±0.52
IR + CAM	RF (3 s)	65.00±0.65	33.93±2.81	29.02±0.89	96.34±0.07	29.81±1.16
	SVM (3 s)	64.07±0.79	24.10±0.98	24.18±0.17	96.17±0.07	22.38±0.23
	MLP (3 s)	65.05±0.66	28.25±3.20	25.40±0.51	96.29±0.06	24.39±0.88
	kNN (3 s)	60.75±1.29	29.91±3.95	26.25±0.90	95.95±0.11	26.54±1.42
IMU + EEG + CAM	RF (1 s)	95.09±0.23	75.52±2.31	66.23±1.11	99.50±0.02	69.36±1.35
	SVM (1 s)	91.16±0.25	66.79±2.79	53.82±0.70	99.07±0.02	55.82±0.77
	MLP (1 s)	94.32±0.31	76.78±1.59	67.29±1.41	99.42±0.03	70.44±1.25
	kNN (1 s)	92.06±0.24	68.82±1.61	58.49±1.14	99.19±0.02	60.51±0.85

## Data Availability

UP-Fall Detection Dataset is publicly available at: http://sites.google.com/up.edu.mx/har-up/. The database website will be provisionally limited from 3 December 2018 to 19 July 2019 due to an open competition that uses this dataset. If interested on using it, we encourage users to contact the correspondence authors for data accessibility during this period.
